# Dietary Copper Intake and Biological Aging Among US Adults, NHANES 2003–2018

**DOI:** 10.1111/acel.70272

**Published:** 2025-10-20

**Authors:** Liujie Zheng, Guoqiang Li, Jingcheng Cao, Zihang Zhao, Liping Zhang, Zhiyong Hou

**Affiliations:** ^1^ Department of Orthopaedic Surgery Hebei Medical University Third Hospital Shijiazhuang Hebei China; ^2^ Department of Physiology, School of Basic Medicine, Hebei Medical University Shijiazhuang Hebei China; ^3^ Engineering Research Center of Orthopedic Minimally Invasive Intelligent Equipment, Ministry of Education Shijiazhuang Hebei China; ^4^ Key Laboratory of Biomechanics of Hebei Province Shijiazhuang Hebei China; ^5^ NHC Key Laboratory of Intelligent Orthopaedic Equipment Shijiazhuang Hebei China

**Keywords:** biological aging, dietary copper intake, dietary inflammatory index, NHANES

## Abstract

While the health effects of dietary copper intake have been widely studied, no research to date has specifically examined its association with biological aging. Here, we aim to explore the relationship between dietary copper intake and biological aging, while examining the mediating role of dietary inflammatory index (DII). This cross‐sectional study included 18,160 adults from the 2003 to 2018 National Health and Nutrition Examination Survey (NHANES). Weighted multivariable linear regression models, subgroup analysis, trend tests, and restricted cubic spline (RCS) were used to analyze the relationship between dietary copper intake and biological aging. Biological aging was measured from different perspectives including phenotypic age (PhenoAge) and phenotypic age acceleration (PhenoAgeAccel). Additionally, mediation analysis explored the mediating role of DII in the above relationships. In this study, we found dietary copper intake was negatively associated with biological aging. Specifically, each 1‐unit increase in dietary copper intake was associated with a 1.12‐year decrease in PhenoAge and a 1.45‐year decrease in PhenoAgeAccel. RCS models revealed a non‐linear relationship between dietary copper intake and biological aging (*p* for nonlinear < 0.001). Specifically, the inverse association was stronger at lower intake levels, with the protective effect plateauing at higher values. Mediation analysis further indicated that DII mediated the above relationships. This study demonstrates a significant negative association between dietary copper intake and biological aging. Public health strategies that increase dietary copper intake may help reduce the burden of biological aging.

## Graphical abstract

In a nationally representative US cohort, greater copper intake correlated with reduced biological aging, but the effect plateaued at higher intakes. The Dietary Inflammatory Index mediated this association, suggesting anti‐inflammatory dietary patterns underlie copper's benefits.

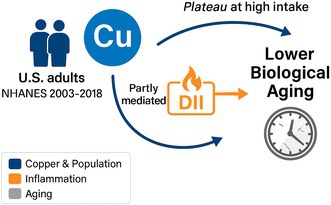

## 1 Introduction

Biological aging is a progressive degenerative process which is expected to become a major global public health challenge in the coming decades (Colchero et al. 2021; Zhang et al. 2022). It involves interconnected changes across multiple biological pathways, ultimately leading to cumulative damage in cells and tissues, functional decline of organs, increased risk of age‐related diseases, and a shortened healthy lifespan (Li et al. 2024; Huang et al. 2025; Prattichizzo et al. 2024). The etiology of biological aging is multifactorial, and its underlying pathological mechanisms are highly complex. Therefore, identifying simple and effective strategies to prevent or delay age‐related pathologies has become a major public health priority, among which dietary intervention may represent a promising direction (Freitas‐Simoes et al. 2016; Wu et al. 2025).

Copper is a crucial trace metal for multiple important biological processes by working as a catalytic and structural cofactor for Cu‐dependent enzymes and a secondary messenger, which is also considered an anti‐inflammatory and antioxidant nutrient (Chen et al. 2020; Bagheri et al. 2021). It cannot be manufactured or stored in the body and must be obtained from daily dietary intake (Hu et al. 2021). Recent studies have suggested that increased dietary copper intake may reduce the risk of several diseases, such as stroke, cognitive decline, gastric cancer, and pelvic inflammatory disease in women (Yang et al. 2022; Meng et al. 2023; Sassano et al. 2024; Hu et al. 2023). Interestingly, we also found that higher copper intake may be associated with a lower risk of biological aging. However, the underlying mechanisms remain unclear. Dietary inflammatory index (DII) is a validated tool used to assess the inflammatory potential of an individual's diet, based on the intake of various nutrients with pro‐ or anti‐inflammatory properties (Shivappa et al. 2014). Previous research has shown that a pro‐inflammatory diet, as indicated by a higher DII score, is associated with an increased risk of biological aging (Wang, Sarker, et al. 2024; Wang, Yan, et al. 2024; Martínez et al. 2023). On the other hand, copper is considered an important anti‐inflammatory nutrient. Based on the above findings, we hypothesize that DII may mediate the negative association between dietary copper intake and biological aging.

Therefore, the aim of our study was to explore the complex relationship between dietary copper intake, DII, and biological aging, with a particular focus on examining the mediating role of DII in the association.

## 2 Materials and Methods

### 2.1 Data Source and Study Population

**FIGURE 1:**
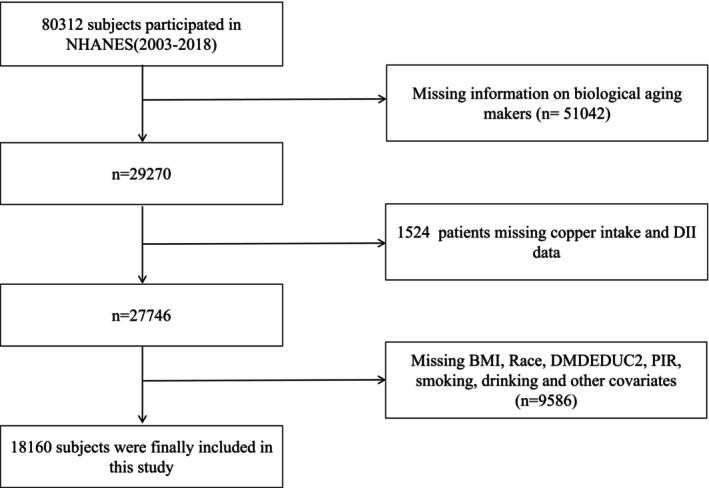
Flowchart of sample selection.

This study utilized data from the NHANES cycles of 2003–2018. Individuals with missing data on key variables including elements required for calculating dietary copper intake, DII, PhenoAge, PhenoAgeAccel, BMI, PIR, education, drinking status, smoking status, hypertension, diabetes, and stroke were excluded from the analysis. The NHANES protocol was approved by the Research Ethics Review Board of the US Centers for Disease Control and Prevention's National Center for Health Statistics. Written informed consent was obtained from all adult participants. This study followed the Strengthening the Reporting of Observational Studies in Epidemiology (STROBE) reporting guideline for cross‐sectional studies. Finally, a total of 18,160 subjects were included in the study (Figure 1).

### 2.2 Exposure and Outcome Variables

Dietary copper intake and DII scores were collected and calculated from dietary intake data which were collected by the Nutrition Methodology Working Group of NHANES via the mean of two reliable 24‐h recall intakes, or a single recall when only one was deemed reliable. In this study, 45 components were selected for the calculation of the DII, including macronutrients, vitamins, minerals, flavonoids, and commonly consumed anti‐inflammatory food components, following the protocol reported by Shivappa et al. (2014). To assess the inflammatory potential of an individual's diet, component‐specific DII scores were summed to yield an overall score: positive values indicate a pro‐inflammatory diet, negative values indicate an anti‐inflammatory diet, and a score of zero reflects a neutral inflammatory potential. Both PhenoAge and PhenoAgeAccel were used to calculate biological aging (Levine et al. 2018). PhenoAge is calculated based on a multivariate mortality risk model that incorporates 10 biomarkers, including chronological age, C‐reactive protein, lymphocyte percentage, white blood cell count, red cell distribution width, alkaline phosphatase, mean cell volume, albumin, creatinine, and glucose. PhenoAgeAccel was defined as the residuals obtained by regressing PhenoAge on chronological age.

### 2.3 Covariates

Potential covariates included demographic, examination, and questionnaire data. Demographic data: age (< 65, ≥ 65), gender (male, female), educational level (under high school, high school or equivalent, college or above), family income (poverty income ratio, low: PIR < 1, medium: 1 ≤ PIR < 3, high: PIR ≥ 3), and race/ethnicity (Mexican American, Other Hispanic, Non‐Hispanic White, Non‐Hispanic Black, other races). Marital status (married, widowed, divorced, separated, never married, living with partner). Examination data: body mass index (BMI, kg/m^2^, normal: BMI < 24, overweight: 24 ≤ BMI < 28, obese: BMI ≥ 28). Physical Activity: total MET minutes per week was calculated according to the methods published before (MacGregor et al. 2021). Participants were categorized into low and high physical activity groups based on whether they met the national physical activity guidelines: low physical activity (< 500 MET‐min/week) and high physical activity (≥ 500 MET‐minutes/week). Questionnaire data: smoking status (yes/no: “yes” defined as having smoked at least 100 cigarettes in a lifetime), alcohol consumption (yes/no: “yes” defined as ever having 4/5 or more drinks every day), hypertension (yes/no: “yes” defined as having ever been told by a health professional that they had high blood pressure or their mean systolic blood pressure (SBP) ≥ 140 mmHg or diastolic blood pressure (DBP) ≥ 90 mmHg), diabetes (yes/no: “yes” defined as having ever been told by a health professional that they had diabetes or FPG ≥ 126 mg/dL (7.0 mmol/L) or 2‐h plasma glucose after the OGTT test (2 h‐PG) ≥ 200 mg/dL (11.1 mmol/L) or A1C ≥ 6.5% or using insulin or taking diabetic pills to lower their blood sugar), stroke (yes/no: “yes” defined as having ever been told by a health professional that they had stroke), and cancer (yes/no: “yes” defined as having ever been told by a health professional that they had cancer).

### 2.4 Statistical Analysis

Data were analyzed using SPSS 26.0 and R software (version 4.2.0), following the guidelines provided by the Centers for Disease Control and Prevention (CDC) (CDC 2001). Given that NHANES employed a complex, multistage probability sampling design to select representative participants, we incorporated primary sampling units, strata, and sample weights in our data analysis. Weighted analyses were conducted using the “survey” package in R statistical software to generate nationally representative estimates. This approach ensured the generalizability of findings to the non‐institutionalized US population while avoiding overestimation of statistical significance. In accordance with NHANES guidelines, weight selection prioritized variables representative of small population subgroups, with appropriate weights applied accordingly. Differences in categorical variables and normally distributed continuous variables were assessed using the chi‐square test and Student's *t*‐test, respectively.

An unadjusted model (Model 1) was first established, followed by a minimally adjusted model (Model 2) that included age, gender, BMI, race, marital status, education, and PIR. A fully adjusted model (Model 3) was subsequently constructed by further including age, gender, BMI, race, marital status, education, PIR, smoking status, hypertension, diabetes, stroke, and physical activity. A *p*‐value < 0.05 was considered statistically significant. Restricted cubic spline (RCS) regression was conducted to assess potential non‐linear associations between dietary copper intake and biological aging. Specifically, we used four knots placed at the 5th, 35th, 65th, and 95th percentiles of the dietary copper intake distribution.

## 3 Results

### 3.1 Participant Characteristics

A total of 18,160 participants met the inclusion criteria. Among them, 8626 (49.50%) were women, and 4010 (16.31%) were aged ≥ 65 years. In terms of race and ethnicity, 2872 (7.64%) participants were Mexican American, 3445 (9.44%) were non‐Hispanic Black, and 8920 (71.98%) were non‐Hispanic White. As shown in Table 1, the general characteristics of participants were summarized according to quartiles of dietary copper intake: Q1 (< 0.81), Q2 (0.81–1.13), Q3 (1.13–1.52), and Q4 (≥ 1.52). Specifically, participants in the highest quartile of copper intake were more likely to be younger, male, non‐Hispanic White, married, and better educated, with higher levels of physical activity and lower poverty‐income ratios. In contrast, those in the lowest quartile tended to be older, female, non‐Hispanic Black, less educated, and more socioeconomically disadvantaged. Moreover, higher copper intake was associated with lower PhenoAge and PhenoAgeAccel values, as well as lower prevalence of hypertension, diabetes, and stroke.

**TABLE 1:** Characteristics of participants based on dietary copper intake.

Characteristic	Total	Q1 (< 0.81)	Q2 (0.81–1.13)	Q3 (1.13–1.52)	Q4 (≥ 1.52)	*p*
	(*n* = 18,160)	(*n* = 5023)	(*n* = 4708)	(*n* = 4396)	(*n* = 4033)	
Age, years						**< 0.001**
	14150 (83.69)	3883 (84.21)	3521 (80.72)	3439 (83.80)	3307 (86.03)	
≥ 65	4010 (16.31)	1140 (15.79)	1187 (19.28)	957 (16.20)	726 (13.97)	
Gender, (%)						**< 0.001**
Male	9534 (50.50)	2176 (39.51)	2220 (43.86)	2443 (52.21)	2695 (66.40)	
Female	8626 (49.50)	2847 (60.49)	2488 (56.14)	1953 (47.79)	1338 (33.60)	
Race/Ethnicity, (%)						**< 0.001**
Mexican American	2872 (7.64)	768 (8.46)	772 (8.10)	711 (7.54)	621 (6.47)	
Other Hispanic	1552 (4.59)	521 (5.64)	397 (4.40)	349 (4.33)	285 (3.99)	
Non‐Hispanic White	8920 (71.98)	2097 (65.01)	2294 (71.44)	2303 (74.87)	2226 (76.58)	
Non‐Hispanic Black	3445 (9.44)	1285 (14.47)	924 (9.79)	708 (7.76)	528 (5.75)	
Other Races	1371 (6.35)	352 (6.42)	321 (6.28)	325 (5.50)	373 (7.21)	
BMI, (%)						**0.020**
	3951 (23.36)	1125 (23.79)	986 (22.93)	895 (22.77)	945 (23.94)	
24–28	4928 (27.48)	1266 (25.37)	1213 (26.69)	1249 (27.98)	1200 (29.88)	
≥ 28	9281 (49.16)	2632 (50.84)	2509 (50.38)	2252 (49.25)	1888 (46.18)	
PIR, (%)						**< 0.001**
	3168 (11.74)	1232 (18.77)	805 (11.04)	632 (9.19)	499 (7.96)	
1–3	7574 (33.58)	2291 (38.79)	2049 (35.16)	1771 (32.22)	1463 (28.14)	
≥ 3	7418 (54.69)	1500 (42.44)	1854 (53.80)	1993 (58.60)	2071 (63.90)	
Marital status, *n* (%)						**< 0.001**
Married	9646 (55.87)	2198 (45.37)	2530 (56.49)	2511 (59.69)	2407 (61.95)	
Widowed	1176 (4.81)	415 (6.08)	346 (5.90)	242 (4.29)	173 (2.96)	
Divorced	2071 (10.70)	651 (12.41)	545 (11.14)	477 (9.51)	398 (9.73)	
Separated	599 (2.42)	228 (3.72)	150 (2.18)	132 (2.14)	89 (1.64)	
Never married	3084 (17.63)	1026 (21.77)	732 (15.54)	691 (17.02)	635 (16.19)	
Living with partner	1584 (8.57)	505 (10.65)	405 (8.75)	343 (7.36)	331 (7.54)	
Education, (%)						**< 0.001**
Less than high school	3842 (12.65)	1396 (17.67)	1059 (13.64)	797 (11.07)	590 (8.22)	
High school or equivalent	4291 (24.00)	1374 (29.66)	1219 (26.30)	946 (21.78)	752 (18.26)	
College or above	10,027 (63.35)	2253 (52.67)	2430 (60.06)	2653 (67.15)	2691 (73.51)	
Physical activity, (%)						**< 0.001**
Low physical activity	6541 (31.75)	2066 (35.17)	1860 (34.92)	1481 (31.34)	1134 (25.60)	
High physical activity	11,619 (68.25)	2957 (64.83)	2848 (65.08)	2915 (68.66)	2899 (74.40)	
PhenoAge, years	56.03 (0.30)	57.32 (0.47)	57.89 (0.43)	55.21 (0.44)	53.69 (0.53)	**< 0.001**
PhenoAgeAccel, years	−1.53 (0.17)	0.91 (0.23)	−0.77 (0.25)	−2.64 (0.21)	−3.64 (0.29)	**< 0.001**
DII scores	0.80 (0.04)	2.18 (0.04)	1.35 (0.04)	0.46 (0.04)	−0.80 (0.04)	**< 0.001**
Alcohol consumption, (%)						0.423
Yes	3007 (15.35)	836 (15.93)	753 (14.87)	710 (14.54)	708 (16.05)	
No	15,153 (84.65)	4187 (84.07)	3955 (85.13)	3686 (85.46)	3325 (83.95)	
Smoking status, (%)						**< 0.001**
Yes	9120 (49.02)	2716 (54.23)	2320 (48.30)	2172 (47.83)	1912 (45.73)	
No	9040 (50.98)	2307 (45.77)	2388 (51.70)	2224 (52.17)	2121 (54.27)	
Cancer, *n* (%)						0.268
Yes	1763 (9.76)	445 (8.84)	490 (10.52)	448 (10.17)	380 (9.53)	
No	16,397 (90.24)	4578 (91.16)	4218 (89.48)	3948 (89.83)	3653 (90.47)	
Hypertension, *n* (%)						**< 0.001**
Yes	7453 (36.27)	2175 (37.22)	2038 (37.63)	1772 (36.63)	1468 (33.63)	
No	10,707 (63.73)	2848 (62.78)	2670 (62.37)	2624 (63.37)	2565 (66.37)	
Diabetes, (%)						**< 0.001**
Yes	3132 (12.55)	959 (13.83)	916 (14.53)	690 (10.97)	567 (10.88)	
No	15,028 (87.45)	4064 (86.17)	3792 (85.47)	3706 (89.03)	3466 (89.12)	
Stroke, (%)						**< 0.001**
Yes	639 (2.52)	233 (3.28)	194 (3.13)	130 (1.99)	82 (1.68)	
No	17,521 (97.48)	4790 (96.72)	4514 (96.87)	4266 (98.01)	3951 (98.32)	

*Note:* Data were presented as weighted means ± SE for continuous variables or *n* (%) for categorical variables.

Abbreviations: BMI, body mass index; DII, dietary inflammatory index; PhenoAge, phenotypic age; PhenoAgeAccel, phenotypic age acceleration; PIR, poverty income ratio.

### 3.2 Association of Dietary Copper Intake With Biological Aging

**FIGURE 2:**
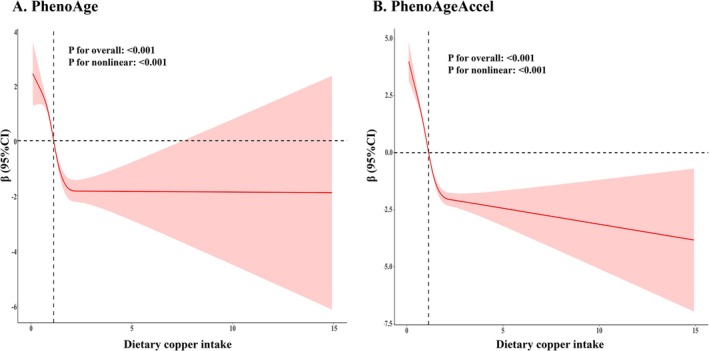
Association of dietary copper intake with biological aging. (A) for PhenoAge and (B) for PhenoAgeAccel. Solid and dashed lines represent the predicted value and 95% CI. Adjusted for age, gender, BMI, race, marital status, education, PIR, smoking status, hypertension, diabetes, stroke, and physical activity. CI, confidence interval.

Both continuous and categorical models were performed to explore the relationship. In the continuous model, the results showed a negative association between dietary copper intake and biological aging markers across all 3 models (Table 2). After adjusting for covariates in Model 3, each 1‐unit increase in dietary copper intake was associated with a 1.12‐year decrease in PhenoAge and a 1.45‐year decrease in PhenoAgeAccel. In the categorical model, compared with the lowest quartiles group, the highest quartiles group exhibited a 3.21‐year decrease for PhenoAge and a 3.97‐year decrease for PhenoAgeAccel, respectively (*p* for trend < 0.001). As shown in Figure 2, RCS analyses showed that the relationships between dietary copper intake and biological aging markers were nonlinear (*p*‐overall < 0.001, *p*‐nonlinear < 0.001). Specifically, the inverse association was stronger at lower intake levels, with the protective effect plateauing at higher values.

**TABLE 2:** Association of dietary copper intake with biological aging.

Characteristics	Model 1		Model 2		Model 3	
	*β* (95% CI)	*p*	*β* (95% CI)	*p*	*β* (95% CI)	*p*
*PhenoAge*						
Copper intake	−1.29 (−1.98 to −0.59)	< 0.001	−1.28 (−1.76 to −0.80)	< 0.001	−1.12 (−1.56 to −0.68)	< 0.001
Q1	Reference		Reference		Reference	
Q2	0.57 (−0.50 to 1.65)	0.298	−0.87 (−1.71 to −0.03)	0.046	−0.72 (−1.47 to 0.03)	0.066
Q3	−2.11 (−3.30 to −0.92)	< 0.001	−2.62 (−3.60 to −1.64)	< 0.001	−2.27 (−3.19 to −1.34)	< 0.001
Q4	−3.64 (−4.86 to −2.42)	< 0.001	−3.76 (−4.73 to −2.79)	< 0.001	−3.21 (−4.09 to −2.34)	< 0.001
*p* for trend	< 0.001		< 0.001		< 0.001	
*PhenoAgeAccel*						
Copper intake	−1.60 (−2.03 to −1.17)	< 0.001	−1.49 (−1.86 to −1.11)	< 0.001	−1.45 (−1.82 to −1.08)	< 0.001
Q1	Reference		Reference		Reference	
Q2	−1.68 (−2.30 to −1.06)	< 0.001	−1.23 (−1.82 to −0.65)	< 0.001	−1.20 (−1.77 to −0.64)	< 0.001
Q3	−3.55 (−4.17 to −2.94)	< 0.001	−3.05 (−3.60 to −2.51)	< 0.001	−2.92 (−3.47 to −2.37)	< 0.001
Q4	−4.55 (−5.19 to −3.90)	< 0.001	−4.12 (−4.70 to −3.54)	< 0.001	−3.97 (−4.54 to −3.40)	< 0.001
*p* for trend	< 0.001		< 0.001		< 0.001	

*Note:* Weighted linear regression models: Model 1: no covariates were adjusted. Model 2 was adjusted for demographic factors, including age, gender, BMI, race, marital status, education and PIR. Model 3 was adjusted for age, gender, BMI, race, marital status, education, PIR, smoking status, hypertension, diabetes, stroke and physical activity.

Abbreviations: PhenoAge, phenotypic age; PhenoAgeAccel, phenotypic age acceleration.

### 3.3 Subgroup Analysis of Dietary Copper Intake and Biological Aging

**FIGURE 3:**
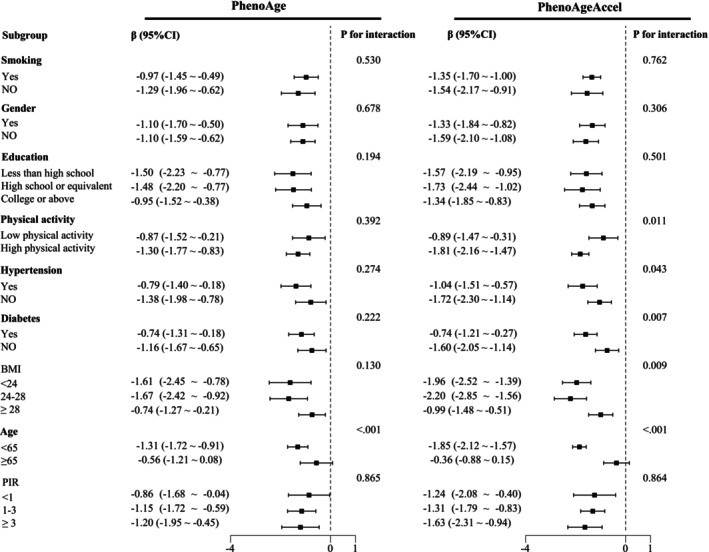
Subgroup analysis of biological aging with dietary copper intake. Adjusted for age, gender, BMI, race, marital status, education, PIR, smoking status, hypertension, diabetes, stroke, and physical activity. CI, confidence interval.

In subgroup analyses, significant interactions (*p* < 0.05) were observed between the dietary copper intake and factors such as age, BMI, physical activity, hypertension, and diabetes (Figure 3). Despite these interactions, the dietary copper intake consistently showed a negative association with biological aging across all subgroups except for those aged 65 or above.

### 3.4 Correlation Between DII and Biological Aging

Both continuous and categorical models were performed to explore the relationship between DII and biological aging. In the continuous model, we found a positive association between DII and biological aging markers across all 3 models (Table 3), each 1‐unit increase in DII was associated with a 0.59‐year increase in PhenoAge and a 0.82‐year increase in PhenoAgeAccel. In the categorical model, compared with the lowest quartiles group, the highest quartiles group exhibited a 3.01‐year increase for PhenoAge and a 3.92‐year increase for PhenoAgeAccel, respectively (*p* for trend < 0.001).

**TABLE 3:** Association of DII with biological aging.

Characteristics	Model 1		Model 2		Model 3	
	*β* (95% CI)	*p*	*β* (95% CI)	*p*	*β* (95% CI)	*p*
*PhenoAge*						
DII scores	0.83 (0.61–1.06)	< 0.001	0.70 (0.51–0.89)	< 0.001	0.59 (0.42–0.77)	< 0.001
Q1	Reference		Reference		Reference	
Q2	1.47 (0.29–2.65)	0.017	0.98 (0.13–1.84)	0.028	0.87 (−0.00–1.73)	0.055
Q3	2.34 (1.23–3.45)	< 0.001	1.55 (0.61–2.48)	0.002	1.25 (0.37–2.13)	0.007
Q4	3.85 (2.69–5.01)	< 0.001	3.49 (2.52–4.47)	< 0.001	3.01 (2.10–3.91)	< 0.001
*p* for trend	< 0.001		< 0.001		< 0.001	
*PhenoAgeAccel*						
DII scores	0.96 (0.84–1.09)	< 0.001	0.85 (0.74–0.96)	< 0.001	0.82 (0.71–0.93)	< 0.001
Q1	Reference		Reference		Reference	
Q2	1.86 (1.25–2.47)	< 0.001	1.44 (0.86–2.02)	< 0.001	1.40 (0.82–1.98)	< 0.001
Q3	2.98 (2.40–3.56)	< 0.001	2.51 (1.97–3.04)	< 0.001	2.44 (1.92–2.96)	< 0.001
Q4	4.61 (3.95–5.27)	< 0.001	4.06 (3.48–4.64)	< 0.001	3.92 (3.35–4.49)	< 0.001
*p* for trend	< 0.001		< 0.001		< 0.001	

*Note:* Weighted linear regression models: Model 1: no covariates were adjusted. Model 2 was adjusted for demographic factors, including age, gender, BMI, race, marital status, education and PIR. Model 3 was adjusted for age, gender, BMI, race, marital status, education, PIR, smoking status, hypertension, diabetes, stroke and physical activity.

Abbreviations: DII, dietary inflammatory index; PhenoAge, phenotypic age; PhenoAgeAccel, phenotypic age acceleration.

### 3.5 Mediating Role of DII

**FIGURE 4:**
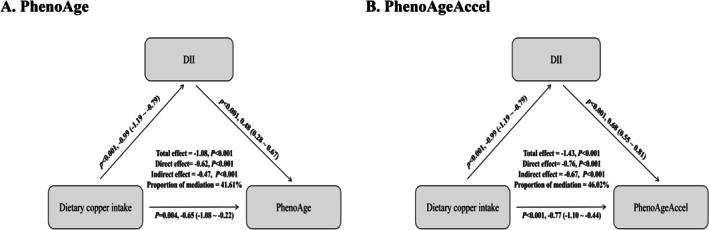
Path diagram of the mediation analysis of DII. (A) for PhenoAge and (B) for PhenoAgeAccel. Adjusted for age, gender, BMI, race, marital status, education, PIR, smoking status, hypertension, diabetes, stroke, and physical activity. CI, confidence interval.

In the mediation analysis, we found that DII scores partially mediated the association between dietary copper intake and both biological aging markers. As shown in Figure 4, the proportion of mediation was 41.61% for PhenoAge and 46.02% for PhenoAgeAccel, respectively. These findings indicate that DII may serve as a key pathway linking copper intake to biological aging.

## 4 Discussion

In this cross‐sectional study based on NHANES 2003–2018 data, we found that higher dietary copper intake was associated with lower levels of biological aging, with the association partially mediated by the DII, even after adjusting for potential confounders. This association was further supported by the results of the subgroup analysis, trend tests, and RCS analysis. To our knowledge, this is the first study to explore the association between dietary copper intake and biological aging, as well as the mediating role of DII.

In the study, we found that dietary copper intake was negatively associated with PhenoAge and PhenoAgeAccel. Both chronic inflammation and oxidative stress have been widely demonstrated to play crucial roles in driving the biological aging process (Meier et al. 2023; Wu et al. 2021; Sebastiani et al. 2017; Zhang et al. 2017). Various supplements and medications with antioxidant and anti‐inflammatory properties have been shown to effectively delay aging, such as salidroside, anthocyanins and their intestinal microbial metabolites, ellagic acid, and lactoferrin (Zhang et al. 2024; Wang, Tang, et al. 2024; Zhu et al. 2022; Li et al. 2022). Copper serves as a cofactor for several enzymes involved in antioxidant defense and the formation of metalloenzymes. An adequate dietary intake of copper may enhance the body's antioxidant and anti‐inflammatory capacities, thereby contributing to the delay of the aging process (Bagheri et al. 2021; Zhang et al. 2023). We also found that DII was positively associated with all biological aging markers, which is consistent with previous studies (Wang, Sarker, et al. 2024; Xie et al. 2023; Hu et al. 2022). Dietary intake, as one of the most important modifiable risk factors influencing human health, has been shown to exert a double‐edged effect on stroke development by regulating immune responses and inflammatory processes. A highly pro‐inflammatory diet, as reflected by higher DII scores, may accelerate aging by increasing serum concentrations of various pro‐inflammatory cytokines, including C‐reactive protein, TNF‐α, IL‐1β, and IL‐6.

Mediation analysis suggests that DII mediated the relationships between dietary copper intake and biological aging. This biological plausibility supports the hypothesis that higher dietary copper intake may reduce the risk of biological aging by modulating systemic inflammation, as reflected by lower DII scores. On the other hand, many of the food items that contribute to dietary copper intake were also included in the DII scoring algorithm, such as whole grains, legumes, nuts, seeds, and leafy green vegetables, which also contribute to the intake of nutrients used to derive DII scores (e.g., fiber, magnesium, omega‐3 fatty acids, and vitamin E). So, DII may act as a proxy mediator, reflecting not only inflammatory status but also a broader anti‐inflammatory dietary pattern commonly associated with copper‐rich diets. The shared dietary sources suggest that copper intake may serve as a marker for a health‐promoting dietary profile, rather than exerting effects in complete isolation. Future studies using dietary pattern analysis or structural equation modeling may further clarify these complex interrelationships.

## 5 Strengths and Limitations

The strengths of this study include its large sample size, the use of nationally representative NHANES data, and the minimization of selection bias through rigorous sampling methodology. However, several limitations should be acknowledged. First, due to its cross‐sectional design, causal relationships cannot be established. Second, dietary copper intake and DII were assessed and calculated using 24‐h dietary recall, which may be subject to recall bias and may not fully represent habitual dietary intake. Further clinical randomized controlled trials are needed to further corroborate our results. Third, there may be some unadjusted potential confounders, such as occupational strain and medication use that were not included in the multivariable models, potentially leading to residual confounding. Fourth, the mediating effect of DII was assessed using cross‐sectional data and regression‐based mediation analysis, which may be vulnerable to temporal ambiguity and model misspecification. Longitudinal mediation models are needed to validate this pathway.

## 6 Conclusion

The findings of this cross‐sectional study suggest that higher dietary copper intake was associated with a lower risk of biological aging with mediation by DII. These results provide insight into the potential role of anti‐inflammatory dietary patterns in guiding interventions to promote healthy aging.

## Author Contributions

Liujie Zheng and Jingcheng Cao collected data and organized the study. Liujie Zheng, Jingcheng Cao, and Zihang Zhao performed the statistical analysis. Liujie Zheng, Guoqiang Li, and Zhiyong Hou drafted the manuscript. Liping Zhang provided important support during the revision process and contributed to the improvement of data analysis and result interpretation. The progressive drafts of the manuscript were reviewed critically, and the final version was approved by all authors.

## Conflicts of Interest

The authors declare no conflicts of interest.

## Data Availability

The data presented in this study are available on request from the corresponding author.
